# Health system delay among patients with tuberculosis in Taiwan: 2003–2010

**DOI:** 10.1186/s12879-015-1228-x

**Published:** 2015-11-02

**Authors:** Chien-Chou Chen, Chen-Yuan Chiang, Sung-Ching Pan, Jann-Yuan Wang, Hsien-Ho Lin

**Affiliations:** Institute of Epidemiology and Preventive Medicine, National Taiwan University, Taipei, Taiwan; Department of Lung Health and NCDs, International Union Against Tuberculosis and Lung Disease, Paris, France; Division of Pulmonary Medicine, Wan Fang Hospital, Taipei Medical University, Taipei, Taiwan; Department of Internal Medicine, College of Medicine, Taipei Medical University, Taipei, Taiwan; Department of Internal Medicine, National Taiwan University Hospital, Taipei, Taiwan

**Keywords:** Tuberculosis, Health system delay, Temporal pattern, Determinants

## Abstract

**Background:**

Taiwan has integrated the previous vertical tuberculosis (TB) control system into the general health care system. With the phase out of the specialized TB care system and the declining TB incidence, it is likely that clinical workers become less familiar with the presentation of TB, resulting in delay in TB diagnosis and treatment.

**Methods:**

We used the detailed information of health care visits in the Taiwan National Health Insurance database to analyze the temporal pattern of the health system delay (HSD) among 3,117 patients with TB between 2003 and 2010.

**Results:**

The median HSD was 29 days (interquartile range 5–73 days), and the median delay increased from 26 days in 2003 to 33.5 days in 2008, thereafter slightly decreased to 32 days in 2010. Patient factors associated with a longer HSD included: aged 45–64 and ≧65 years (as compared to aged <30 years); females (as compared to males); an initial visit as an outpatient (as compared to an inpatient). Provider factors were an initial visit to a provider not specialized in TB (as compared to a TB-related provider), to a primary care clinic or to a medical center (as compared to a district hospital), and in Central region, Northern region, KaoPing region, Southern region and Taipei region (as compared to in Eastern region). Longer distances from the point of initial visit to that of treatment were associated with longer HSD. Patients who switched among different levels or different types of medical care services during their illness exhibited the longest HSD.

**Conclusions:**

In countries where the TB care systems are being restructured from a vertical to a horizontal system, it is critical to monitor HSD and be aware of its increase. The potential increase in the HSD from 2003 to 2008 observed in this study is concerning and the decline of HSD after 2008 might be attributed to the launch of contact investigation. Our results call for actions to improve the efficiency of TB diagnosis in the health care system and to increase the awareness of TB among physicians and the general public.

**Electronic supplementary material:**

The online version of this article (doi:10.1186/s12879-015-1228-x) contains supplementary material, which is available to authorized users.

## Background

Tuberculosis (TB) remains a major cause of morbidity and mortality among all communicable diseases in Taiwan [1]. Although the incidence and mortality of TB in Taiwan have been decreasing, the decline rate has slowed down in recent years [2, 3]. TB case finding in Taiwan mainly depends on the identification of symptomatic patients presenting themselves at health care facilities, supplemented by contact tracing and active screening in high risk groups. Because of the non-specific clinical manifestations of TB, health care workers might fail to identify patients with presumptive TB, resulting in substantial delay in the diagnosis and treatment of TB and an increased risk of transmission [4, 5].

Since the health sector reform in 2002, Taiwan has integrated the previous vertical TB control system into the general health care system [6]. With the phase out of the specialized TB care system and the declining TB incidence, it is likely that clinical workers become less familiar with the presentation of TB [7]. As a result, the interval from the initial medical consultation of a patient to the treatment of TB (health system delay, HSD) [8, 9] could have increased in Taiwan over the past years. Meanwhile, if the general health care system is not efficient in promptly identifying patients with TB, the patients might need to make repeated visits within the health care system for a prolonged period before being diagnosed with TB [10]. Using a cohort of new TB patients, we investigated the temporal pattern of HSD and its determinants in Taiwan between 2003 and 2010.

## Methods

### Study population

The study population included all patients with incident TB between 2003 and 2010 from the cohort of Longitudinal Health Insurance Database 2000 (LHID2000) (http://nhird.nhri.org.tw/en/index.html). To ensure the representativeness, the research database of LHID2000 contains all the original claims data of a total of 1,000,000 individuals who were randomly sampled from the 23 million beneficiaries enrolled between 1996 and 2000 in the National Health Insurance (NHI) programme [11]. LHID2000 contains all the registration and claims data including utilization of inpatient and outpatient services by the 1,000,000 individuals. The NHI covers more than 98 % of the total population in Taiwan [12]. The four levels of health care system in Taiwan, from the lower level to the higher level, are local clinic, district hospital, regional hospital, and medical center (tertiary medical service). People can choose to go to all four levels of health care facilities directly, and can be seen by non-specialists or TB specialists under the current universal health insurance. The study was approved by the ethics committee of the National Taiwan University. Since the data on patient identities were scrambled cryptographically to protect privacy, informed consent was exempted by the committee.

### Measurement of active tuberculosis

Patients were classified as having incident TB if they met the following criteria in the LHID2000 during the follow-up period (2003–2010): (1) at least one medical visit with TB diagnosis based on the International Classification of Diseases, 9th Revision, Clinical Modification (ICD-9-CM) codes 010–018; (2) prescriptions of at least two anti-TB medications for ≥28 days [13, 14] (Additional file 1: Figure S1). According to the national guidelines on TB diagnosis and treatment in Taiwan, the diagnosis of active TB was based on physical examination, chest radiograph, bacteriological evidence from sputum smear and culture, and a positive response to anti-TB treatment [15]. Laboratory facilities that are able to perform sputum smear or culture examination for *Mycobacterium tuberculosis* are widely accessible throughout Taiwan [16].

### Validation of TB case definition

We used an independent cohort to validate the TB case definition in the study. Participants of the 2005 National Health Interview Survey (NHIS) (http://nhis.nhri.org.tw/) were included in the cohort [17]. The data in the NHIS cohort (*n* = 18,526) were cross matched with those in the NHI database and the TB registry of Taiwan Centers for Disease Control (CDC). The incident TB case in the NHI database was identified based on the previously mentioned ICD-9-CM codes and the prescriptions of two anti-TB medications for ≥28 days (NHI-based), whereas the confirmation of TB in the TB registry was based on bacteriological evidence and clinical information [15]. Assuming the notification in the TB registry was the gold standard, we estimated that the NHI-based TB definition in the 2005 NHIS has a sensitivity and specificity of 87.9 and 99.9 %, respectively. When the anti-TB treatment duration was changed from ≥28 to ≥56 days, the sensitivity and specificity of TB became 86.9 and 99.9 %, respectively.

### Measurement of health system delay

In this study, we defined HSD as the interval between the first medical consultation of respiratory-related diseases and the initiation of TB treatment. For each patient with TB, we extracted claims data associated with the respiratory-related visits from the LHID2000 according to the ICD-9-CM codes, prescriptions, and medical procedures pertaining to respiratory diseases (Additional file 2: Table S1, Additional file 3: Table S2 and Additional file 4: Table S3). To identify the first medical consultation of a TB patient, two sequential respiratory-related visits were considered belonging to the same respiratory episode if the interval between the two visits was ≤60 days, and different respiratory episodes if the interval was >60 days [18] (Additional file 5: Figure S2). The observation window for measuring HSD was 15 months before the start of TB treatment. We excluded patients with chronic respiratory diseases from the analysis. Such patients were likely to have frequent respiratory-related visits and therefore their HSD could not be determined. Chronic respiratory diseases were defined based on prescriptions of respiratory medicines for ≥28 days at the baseline (365–730 days before the initiation of TB treatment). Similarly, patients with asymptomatic TB or extra-pulmonary TB were not included in this analysis because of the working definition of HSD.

### Statistical analysis

To examine the pattern of HSD from 2003 to 2010, we plotted the median and interquartile range (IQR) of HSD by year and visually inspected the trend. We investigated patient and provider factors as the HSD determinants [6, 8, 9]. Patient factors were age group, sex, monthly salary, and patient type (outpatient vs. inpatient); provider factors were the specialty type of the first consultation (TB-related, including Chest, Chest Surgery, TB, Infectious Disease, and Pulmonary and Critical Care vs. not specialized in TB), the level of medical care service (primary care clinic, district hospital, regional hospital, and medical center) where the first consultation was received, and the provider location of the initial visit (Central, Eastern, KaoPing, Northern, Southern, and Taipei regions). One continuous variable, care-seeking distance (the Euclidean distance between the point of the first consultation and that of the initiation of TB treatment), was included. Because providers’ location information was only available at the township level, each provider was assigned the respective township centroid (XY coordinates) to approximate the exact location [19].

We treated HSD as time to event data and investigated the HSD determinants by using survival analysis. Cox proportional hazards regression was used to estimate the hazard ratios (HRs) of determinants in both univariable and multivariable models [20]. Observations in which the HSD was zero days were randomly assigned a tiny value (0.01–0.05) to approximate the true model, in which a fraction of patients had a zero survival time. An HR <1 indicated that the determinant was associated with a longer HSD and vice versa.

We further examined a patient’s care-seeking pathways from the first medical consultation to the initiation of TB treatment. We marked the patient’s visits in chronological sequence according to the specialty type and medical care level, respectively. For example, if a patient sought three primary care clinics during the TB episode, his or her care-seeking pathways would be marked as clinic-- > clinic-- > clinic. We classified care-seeking pathways as “persistent” if the patient continuously visited the same specialty type or medical care level of provider during the TB episode; “progressive” if the patient switched from a provider not specialized in TB to a TB-related provider, or from a primary care provider to a higher level of medical care during the study interval; “mixed” if the pattern was neither “persistent” nor “progressive”. We used SAS 9.3 (SAS Institute Inc., Cary, NC, USA) to perform the statistical analysis and ArcGIS 10.2 (ESRI, Redlands, CA, USA) to measure the care-seeking distance.

## Results

**Fig. 1:**
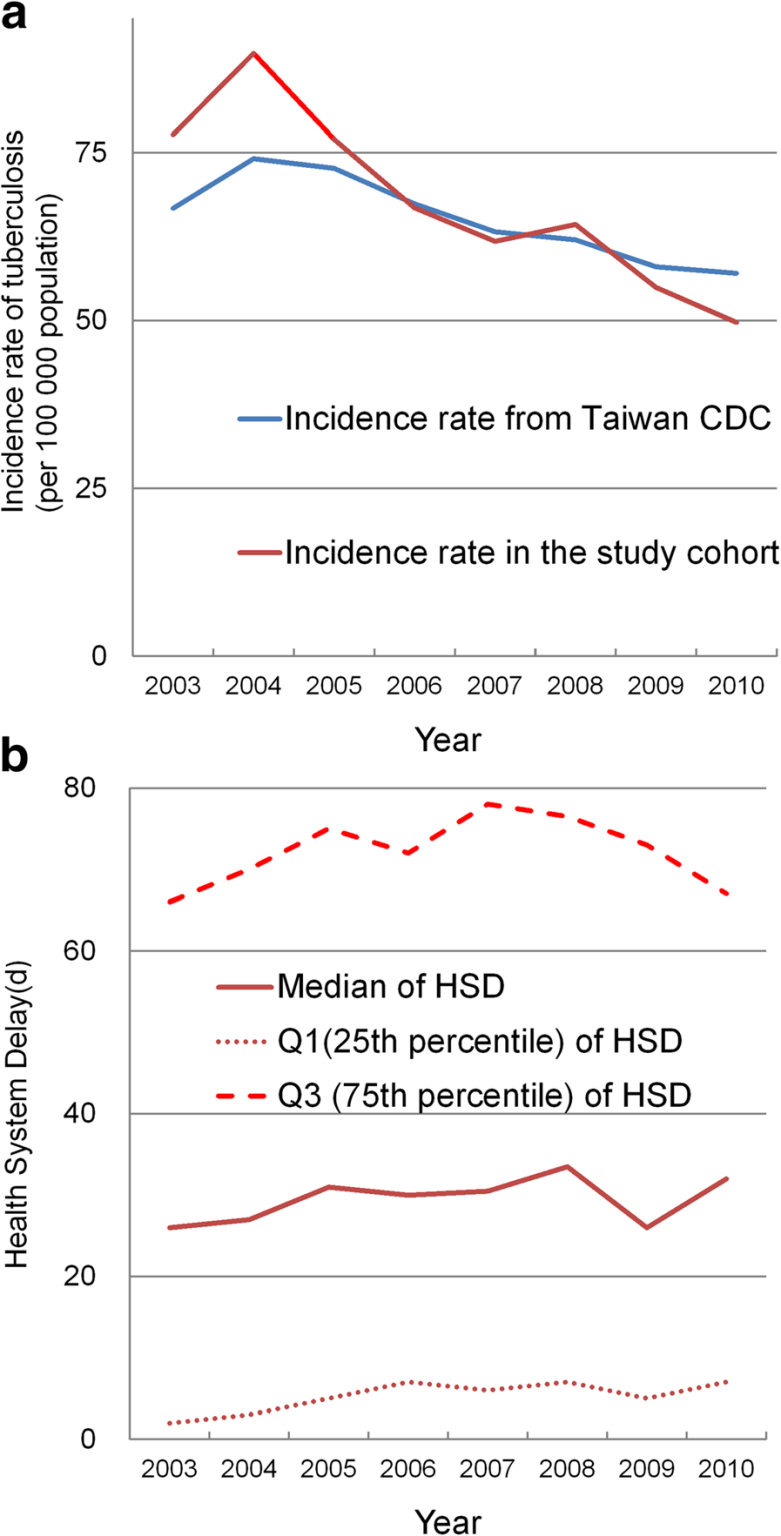
Incidence rate of tuberculosis (TB) and health system delay (HSD): 2003–2010, Taiwan. **a** Annual TB incidence rate observed in the study cohort (red line) compared with that reported by the Taiwan Centers for Disease Control (CDC) (blue line), 2003–2010; **b** median HSD (solid line) and the corresponding Q1 (25th percentile) and Q3 (75th percentile) of HSD (dashed lines) according to calendar year

A total of 4,494 patients with incident TB were identified in the LHID2000 cohort between 2003 and 2010. The trend of the TB incidence rate in our cohort is similar to that reported by the Taiwan CDC (Fig. 1a). After excluding 1,377 patients who had a history of chronic respiratory diseases before the TB episode, 3,117 patients were included in the final analysis. The HSD in these patients was right skewed, with a median of 29 days (IQR 5–73; range 0–449). Among the 3,117 patients, 520 (17 %) had a HSD of zero days (i.e., TB treatment was initiated in the first medical consultation of respiratory-related diseases). Overall, the median HSD increased from 26 days in 2003 to 33.5 days in 2008, thereafter slightly decreased to 32 days in 2010 (Fig. 1b). A sensitivity analysis conducted according to different cut-off intervals (30 and 45 days) between two respiratory-related visits revealed a similar HSD trend (Additional file 6: Figure S3).

The median HSD differed by age group, sex, monthly salary, patient type, specialty type, and medical care level (Table 1). Patients who first presented to primary care clinics had substantially long HSDs (median 52 days, IQR 18–100). In the multivariable survival (time to treatment) analysis, patient factors associated with a longer HSD included aged 45–64 years (adjusted HR = 0.81, 95 % confidence interval [CI] 0.72–0.91) and aged ≧65 years (adjusted HR = 0.68, 95 % CI 0.62–0.76) as compared to aged <30 years; females (adjusted HR =0.88, 95 % CI 0.82–0.96) as compared to males; an initial visit as an outpatient (adjusted HR = 0.54, 95 % CI 0.48–0.60) as compared to an inpatient. Provider factors included an initial visit to a provider not specialized in TB (adjusted HR = 0.47, 95 % CI 0.42–0.52) as compared to a TB-related provider, to a primary care clinic (adjusted HR = 0.75, 95 % CI 0.67–0.83) or to a medical center (adjusted HR = 0.79, 95 % CI 0.69–0.90) as compared to a district hospital, and in Central region (adjusted HR = 0.83, 95 % CI 0.69–0.99), Northern region (adjusted HR = 0.71, 95 % CI 0.59–0.86), KaoPing region (adjusted HR = 0.79, 95 % CI 0.66–0.94), Southern region (adjusted HR = 0.75, 95 % CI 0.62–0.90) and Taipei region (adjusted HR = 0.80, 95 % CI 0.67–0.95) as compared to in Eastern region. The longer distance traveled from the point of initial visit to that of treatment was associated with a longer HSD (adjusted HR = 0.96, 95 % CI 0.95–0.97) (Table 2). Patients from Eastern region had traveled the longest distance to be cured (Additional file 7: Figure S4).

**Table 1:** Characteristics of patients with tuberculosis (TB) and distribution of health system delay (HSD) (*n* = 3117)

Variable	*n* (%)		Median HSD (IQR)^a^		*P* value^c^
Age					<0.001
< 30	466	(15.0)	20.5	(2.0–61.0)	
≥ 30 and <45	554	(17.8)	26.0	(4.0–65.0)	
≥ 45 and <65	957	(30.7)	28.0	(5.0–72.0)	
≥ 65	1140	(36.5)	36.0	(7.0–85.0)	
Sex					<0.001
Female	952	(30.5)	35.5	(7.0–76.0)	
Male	2165	(69.5)	26.0	(4.0–70.0)	
Salary					0.010
< $17880 NTD^d^ per month	1811	(58.1)	27.0	(4.0–70.0)	
≥ $17880 NTD per month	1306	(41.9)	32.0	(7.0–75.0)	
Patient type^b^					<0.001
Inpatient	427	(13.7)	0	(0–24.0)	
Outpatient	2690	(86.3)	35.0	(8.0–78.0)	
Specialty type^b^					<0.001
Non-TB-related	2443	(78.3)	40.0	(11.0–85.0)	
TB-related^e^	674	(21.6)	1.0	(0–24.0)	
Medical care level^b^					<0.001
Medical center	482	(15.4)	14.0	(0–47.0)	
Regional hospital	706	(22.6)	10.0	(0–45.0)	
District hospital	488	(15.7)	16.0	(1.0–52.0)	
Clinic	1441	(46.2)	52.0	(18.0–100.0)	
Region^b^					0.050
Central	532	(17.1)	30.0	(6.0–77.0)	
Eastern	150	(4.8)	14.0	(1.0–53.0)	
KaoPing	631	(20.2)	29.0	(5.0–75.0)	
Northern	379	(12.1)	32.0	(5.0–72.0)	
Southern	501	(16.1)	29.0	(5.0–73.0)	
Taipei	924	(29.6)	30.0	(7.0–70.5)	

^a^Interquartile range

^b^Initial visit

^c^Kruskal-Wallis test

^d^17 880 NTD (New Taiwan Dollar) is the monthly minimum wage in Taiwan

^e^Chest, Chest Surgery, TB, Infectious Disease, and Pulmonary and Critical Care

**Table 2:** Cox proportional hazards analysis on health system delay (HSD) for patients with tuberculosis (TB) in Taiwan (*n* = 3115)^e^

Variable	Number	Person-years	Univariable		Multivariable^c^	
			HR (95 % CI)	*P* value	HR (95 % CI)	*P* value
Age						
< 30	466	53.2	1.00		1.00	
≥ 30 and <45	554	75.5	0.87 (0.77–0.98)	0.030	0.89 (0.79–1.00)	0.050
≥ 45 and <65	957	135.9	0.85 (0.76–0.95)	0.005	0.81 (0.72–0.91)	<0.001
≥ 65	1138	198.8	0.72 (0.65–0.80)	<0.001	0.68 (0.62–0.76)	<0.001
Sex						
Female	952	152.8	0.91 (0.84–0.98)	0.021	0.88 (0.82–0.96)	0.003
Male	2163	310.8	1.00		1.00	
Salary						
< $17880 NTD^a^ per month	1810	261.7	1.00		1.00	
≥ $17880 NTD per month	1305	201.9	0.94 (0.87–1.00)	0.080	1.00 (0.93–1.08)	0.901
Patient type^b^						
Inpatient	427	26.9	1.00		1.00	
Outpatient	2688	436.6	0.44 (0.39–0.49)	<0.001	0.54 (0.48–0.60)	<0.001
Specialty type^b^						
Non-TB-related	2441	429.5	0.36 (0.33–0.40)	<0.001	0.47 (0.42–0.52)	<0.001
TB-related^d^	674	34.1	1.00		1.00	
Medical care level^b^						
Medical center	481	46.8	1.09 (0.96–1.23)	0.170	0.79 (0.69–0.90)	<0.001
Regional hospital	706	64.9	1.15 (1.02–1.29)	0.010	0.89 (0.79–1.01)	0.079
District hospital	488	53.0	1.00		1.00	
Clinic	1440	298.8	0.58 (0.52–0.64)	<0.001	0.75 (0.67–0.83)	<0.001
Region^b^						
Central	532	80.5	0.79 (0.66–0.95)	0.010	0.83 (0.69–0.99)	0.045
Eastern	150	17.2	1.00		1.00	
KaoPing	630	98.7	0.77 (0.65–0.92)	0.005	0.79 (0.66–0.94)	0.010
Northern	379	59.1	0.78 (0.64–0.94)	0.010	0.71 (0.59–0.86)	<0.001
Southern	501	77.8	0.79 (0.66–0.95)	0.010	0.75 (0.62–0.90)	0.002
Taipei	923	130.1	0.83 (0.70–0.99)	0.040	0.80 (0.67–0.95)	0.012
Distance (logarithm)	-	-	0.94 (0.930.94)	<0.001	0.96 (0.95–0.97)	<0.001

^a^$17 880 NTD (New Taiwan Dollar) is the monthly minimum wage in Taiwan

^b^Initial visit

^c^Adjusted for all other variables in the table

^d^Chest, Chest Surgery, TB, Infectious Disease, and Pulmonary and Critical Care

^e^Two patients whose care visits (initial or the initiation of TB treatment) outside the island of Taiwan were excluded

Of the 3,117 patients with TB, a total of 16,419 health care visits were made before the initiation of TB treatment (5.2 visits per patient prior to treatment). Furthermore, 28 % of the patients visited the same specialty type (TB-related vs. not specialized in TB) throughout the care-seeking process, 47 % shifted from the non-TB-related to the TB-related specialty, and 25 % exhibited a mixed pattern. Additionally, 26 % opted for the same medical care level, 39 % progressed to a higher level, and 35 % exhibited a mixed pattern (Table 3). Patients with the mixed pattern exhibited the longest HSD (median 63 and 76 days, for the mixed specialty type and the mixed medical care level, respectively).

**Table 3:** Patterns of care-seeking pathways and health system delay (HSD) for patients with tuberculosis (TB) in Taiwan according to specialty type and medical care level of provider (*n* = 2597)

Specialty type: TB-related^a^(T) versus non-TB-related (NT)			Medical care level: primary care clinic (C); district hospital (D); regional hospital (R); medical center (M)		
Pattern	*n* (%)	Median HSD^e^ (IQR)	Pattern	*n* (%)	Median HSD^e^ (IQR)
1.Persistent^b^			1.Persistent^b^		
NT to NT	511 (19.7)	39.0 (13.5–78.0)	C to C	82 (3.1)	23.5 (7.5–61.0)
T to T	215 (8.2)	14.0 (7.0–37.0)	D to D	119 (4.5)	18.0 (7.0–45.0)
			R to R	269 (10.4)	19.0 (7.0–44.0)
			M to M	220 (8.5)	19.5 (7.0–42.0)
2.Progressive^c^	1223 (47.2)	39.0 (13.0–77.0)	2.Progressive^c^	1001 (38.7)	36.0 (13.0–69.0)
3.Mixed^d^	648 (24.9)	63.0 (31.0–128.3)	3.Mixed^d^	906 (34.8)	76.0 (38.0–137.8)

^a^Chest, Chest Surgery, TB, Infectious Disease, and Pulmonary and Critical Care

^b^Patients continuously visited the same specialty type or level of provider during the TB episode

^c^Patients switched from non-TB-related provider to TB-related provider or from primary care provider to higher level of medical care during the care-seeking process

^d^Neither “persistent” nor “progressive”

^e^ *P* value <0.001 (Kruskal-Wallis test)

## Discussion

To our knowledge, this study is the first to characterize HSD temporally by using detailed claims data of a population-based cohort. The results suggested that the HSD could have increased during 2003–2008 in Taiwan. The analysis of HSD determinants further indicated that old age, being female, and outpatients were important patient factors for HSD; provider factors included primary care clinic, medical center, provider not specialized in TB, care-seeking distance, and location in Central, Northern, Southern, KaoPing and Taipei regions. Patients with the mixed pattern of care-seeking pathways exhibited the longest HSD.

HSD can be divided into three interconnected components: a) the interval between the first medical consultation and the time when a TB diagnostic test is ordered; b) the interval between the order of diagnostic test and a positive result (i.e., turnaround time of diagnostic test); c) the interval between the positive diagnostic test and the initiation of TB treatment. Although we were not able to analyze the relative contribution of the three components to the overall HSD, we suspected that the contribution of b and c might be small. High quality TB diagnosis and free TB treatment are widely available in Taiwan [16]. Therefore, the long HSD in our study may be possibly due to the long interval between the first medical consultation and the time when TB is suspected. However, a definitive analysis is still warranted.

The potential increase in HSD between 2003 and 2008 (Fig. 1b) may be a warning message for TB control in Taiwan. Physicians might have fewer experiences in handling TB cases because of the continuous decline in the incidence of TB [3, 7]. The increasing proportion of elderly patients with TB might have also contributed to the HSD increase over time [21]. Nonetheless, we note that the median HSD started to decline after 2008, and the third quartile of HSD declined since 2007, suggesting that the number of patients with extreme HSD was falling (Fig. 1b). One possible reason for the decline was the effect of contact investigation. Taiwan CDC has strengthened the effort of contact investigation since 2007, and the number of close contacts under investigation increased from 2.6 per index case in 2006 to 6.6 per index case in 2010 [22]. Our analysis suggested that routinely collected information can be used to continuously monitor the trend of HSD and to inform policy making. In the meanwhile, alertness on the presentation of TB should be emphasized in the continual medical education of all physicians.

We found that 17 % of patients have 0 days of HSD, suggesting that patients were treated on the same day based on symptoms and results of chest X-ray. In Taiwan, chest X-ray is widely available as an initial test for TB diagnosis [15]. In a previous study conducted in Taiwan, 46 % of 1,126 newly diagnosed TB patients were put on anti-TB treatment immediately based solely on the result of chest X-ray [23]. We caution that same-day treatment based on TB symptoms and X-ray might result in substantial over-treatment, because TB symptoms and chest X-ray tend to have low specificity [24]. Further studies are needed to identify and quantify the situation of over-treatment in Taiwan.

The HSD estimate derived in this study is longer than the result of Chiang et al. (median HSD 23 days) [6] and shorter than that of Lai (median HSD 53 days) [18]. The study by Chiang et al. in 2003 was based on interview of patients with TB in southern Taiwan, and therefore might be subject to recall bias. However, the study by Lai was a population-based study in 2005 analyzing claims data of Taiwanese patients and was unlikely to miss relevant patient visits. The difference between the HSD values estimated in the current study and that obtained by Lai might be attributed to our exclusion of patients with TB who had chronic respiratory diseases at the baseline. The exclusion was necessary because it was not possible to estimate the delay duration in these patients with chronic respiratory diseases by using a claims database. Compared with other countries with an intermediate TB disease burden, the HSD in Taiwan remains long. For example, the median HSD was 15 days in Croatia [25], whereas that was 29 days in Taiwan. Similar to Taiwan, Croatia provides universal health insurance coverage to the whole population. But unlike Taiwan, the primary care physicians in Croatia serve as the gatekeepers to access specialized ambulatory care [26]. Therefore the differences in HSD in the two countries might be partly due to different referral systems.

We identified potential patient and provider factors of HSD. First, the delay increases as patients become older (Table 2). Elderly outpatients might present with non-specific or mild symptoms, making it difficult for general practitioners to diagnose TB [27, 28]. Female outpatients might seek medical attention at an earlier stage of disease (i.e., having a shorter patient delay compared to male patients) and the diagnosis of early-stage TB could be more challenging [25, 29]. Our results are consistent with previous studies that an initial visit to a private practitioner [28, 30] or to a specialty not specialized in TB [31, 32] is a risk factor for HSD. Lastly, we found that the HSD was longer in medical centers than in district hospitals. One possible explanation is that the proportion of patients with comorbidity which made diagnosis of TB difficult was higher in medical centers than in district hospitals under the current universal health insurance in Taiwan [33].

We also observed the regional differences of HSD [31, 34] in Taiwan. Patients from Eastern region had a surprisingly short delay. We further analyzed the health system-level characteristics that might affect the HSD differences among regions. We found that the density of medical providers, in particular the TB-related providers, was associated with HSD differences among regions (Additional file 8: Table S4). The regions with the highest density of TB-related providers (Eastern and Central) were the regions with the shortest HSD in the multivariable analysis (Table 2). Additional studies are warranted to understand the exact reasons for the difference.

In the analysis of the care-seeking pathways, a substantial proportion (25–35 %) of patients with TB exhibited a mixed (neither “persistent” nor “progressive”) pattern (Table 3). This suggests that patients were switching among different levels or different types of medical care services during their illness. The “doctor shopping” behavior [35] has been documented among patients with upper respiratory tract infection, partly due to the ease of accessibility and affordability of medical care in Taiwan. The HSD among patients exhibiting the mixed type of care seeking pattern was almost twice as long as that of patients in the other groups. A better understanding and characterization of patients exhibiting the mixed care-seeking behavior might help to decrease the HSD in Taiwan. The results of spatial analysis indicated that a longer distance travel from the point of initial consultation to that of treatment was associated with a longer HSD. Patients seeking care before TB diagnosis might spread the disease when traveling [36]. To reduce the risks of transmission during care seeking, the referral mechanism must be strengthened [6].

The current study has some limitations. First, we did not measure patient delay (the delay from the onset of illness to the time of initial consultation). Therefore, the interpretation of HSD determinants should be cautious. As mentioned, female Taiwanese might have a shorter patient delay because they are more health conscious than males, and may present themselves to the health care setting at the early stage of disease [35]. Because it is more difficult to diagnose early-stage TB, the female patients may appear to have a longer HSD. Further studies that simultaneously measure patient delay and HSD would be most informative. Second, we used a cut-off interval of 60 days for distinguishing two respiratory episodes. Although the sensitivity analysis conducted using different cut-off intervals (30 and 45 days) revealed similar HSD trends, additional studies are required to validate the assumption. Third, because the LHID2000 contains only the medical claims data, information on whether TB was microbiologically confirmed or not was not available from the database. Fourth, we excluded patients with asymptomatic TB or extra-pulmonary TB based on the current HSD definition. Furthermore, patients with chronic respiratory diseases were excluded from the analysis. Thus, the derived results might not be generalizable to patients with chronic respiratory diseases who may have a longer or shorter HSD than that of the study’s population. However, this does not affect the internal validity of the analysis conducted on those without chronic respiratory diseases. Additionally, the operational definition of chronic respiratory diseases must be validated further in the future. Finally, we didn’t estimate the HSD in recent years (2011 ~ 2014) because of lack of access to more recent claims data.

## Conclusions

The potential increase in the HSD from 2003 to 2008 observed in this study is concerning and it suggested that the health care system might become less vigilant about TB during the period of health sector reform. On the other hand, our historical analysis also revealed that HSD might be shortened through intensified efforts such as contact tracing. Our findings suggest that in countries where the TB care systems are being restructured from a vertical to a horizontal system, it is critical to monitor HSD and be aware of its increase. Actions need to be taken to improve the efficiency of TB diagnosis in the health care system and to increase the awareness of TB among physicians and the general public. In the meanwhile, active case finding including contact investigation and screening of high risk population might help reduce the delay in TB diagnosis and treatment [37, 38]. Further studies should be undertaken to continuously monitor the HSD and to examine the causes of the mixed care-seeking pattern.

## Additional files

Additional file 1: Figure S1 The overall procedure of the study. (PDF 281 kb) Additional file 2: Table S1 ICD-9-CM codes associated with respiratory-related visits. (PDF 191 kb) Additional file 3: Table S2 Medicines associated with respiratory-related visits. (PDF 86 kb) Additional file 4: Table S3 Procedures associated with respiratory-related visits. (PDF 161 kb) Additional file 5: Figure S2 Measurement of health system delay (HSD). We assumed that respiratory-related visits were of the same respiratory episode if the interval between two respiratory-related visits was ≦60 days and different episodes if >60 days when examining HSD. For example, Visit 3 was considered belonging to the same episode and included in our study because the interval between Visit 3 and Visit 4 was 35 days (≦60). Visit 2 was identified as another respiratory episode and excluded because the interval between Visit 2 and Visit 3 was 66 days (>60). $\overline{AB}$ indicates the Euclidean distance between Visit 3 and the visit where TB treatment was initialized (Visit 5). (PDF 311 kb) Additional file 6: Figure S3 Sensitivity analysis of health system delay (HSD) according to different cut-off intervals between two respiratory-related visits. (PDF 168 kb) Additional file 7: Figure S4 Travel distance from the point of initial visit to that of the initiation of TB treatment by region. (PDF 104 kb) Additional file 8: Table S4 Number of medical providers and tuberculosis (TB)-related providers by region in 2010. (PDF 164 kb)

## Abbreviations

TB: Tuberculosis
HSD: Health system delay
LHID2000: Longitudinal health insurance database 2000
NHI: National Health Insurance
ICD-9-CM: International classification of diseases, 9th Revision, clinical modification
NHIS: National Health Interview Survey
CDC: Centers for disease control
IQR: Interquartile range
HRs: Hazard ratios
CI: Confidence interval
