# Supplementary figures and images for: Health system delay among patients with tuberculosis in Taiwan: 2003–2010
Source: BMC Infect Dis. 2015 Nov 2;15:491. doi: 10.1186/s12879-015-1228-x (PMC4629405; doi:10.1186/s12879-015-1228-x)

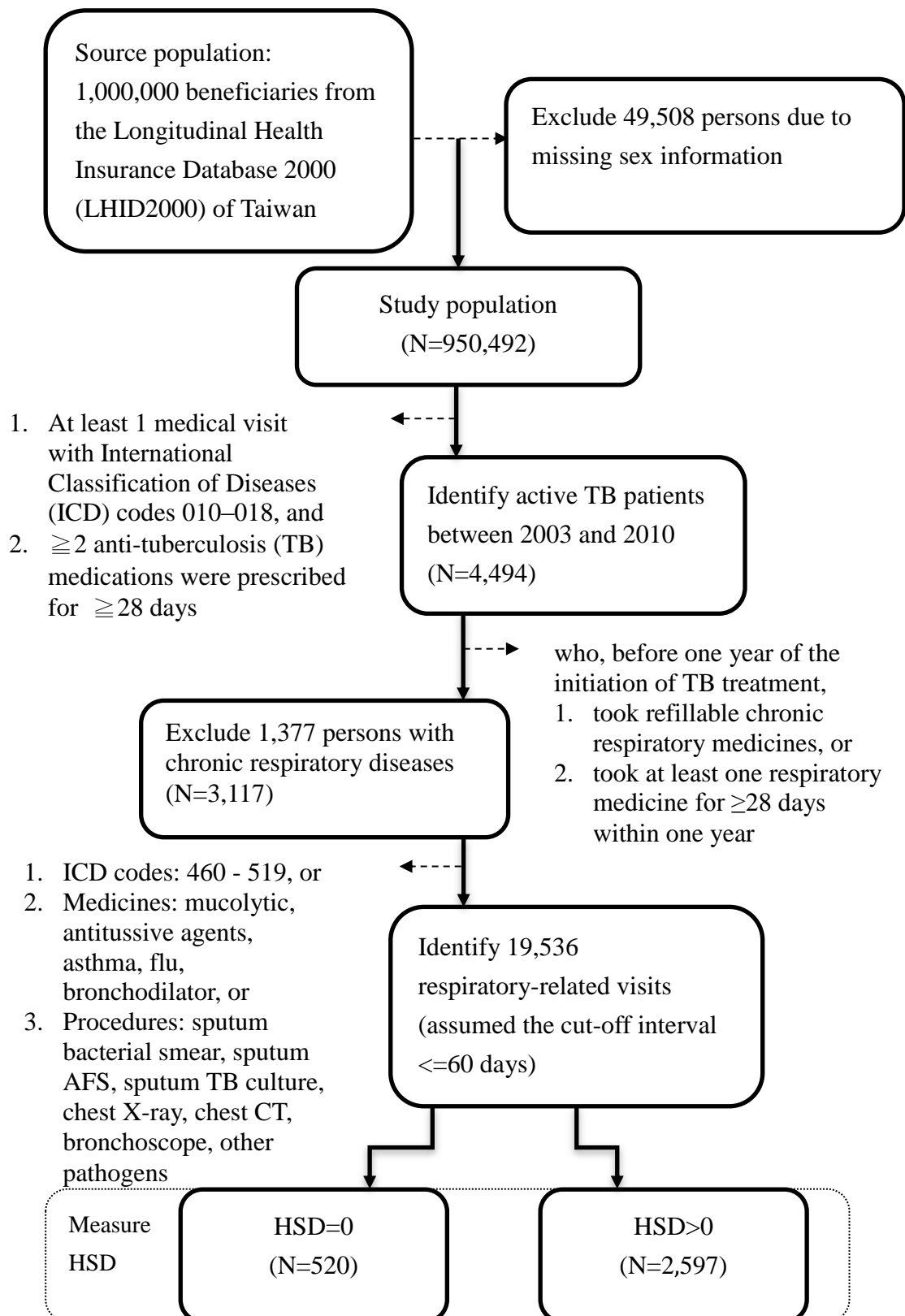

Supplement: Additional file 1: Figure S1 — The overall procedure of the study. (PDF 281 kb) [file 12879_2015_1228_MOESM1_ESM.pdf]

Other disease process

$$\text{HSD} = 35 + 29 = 64$$

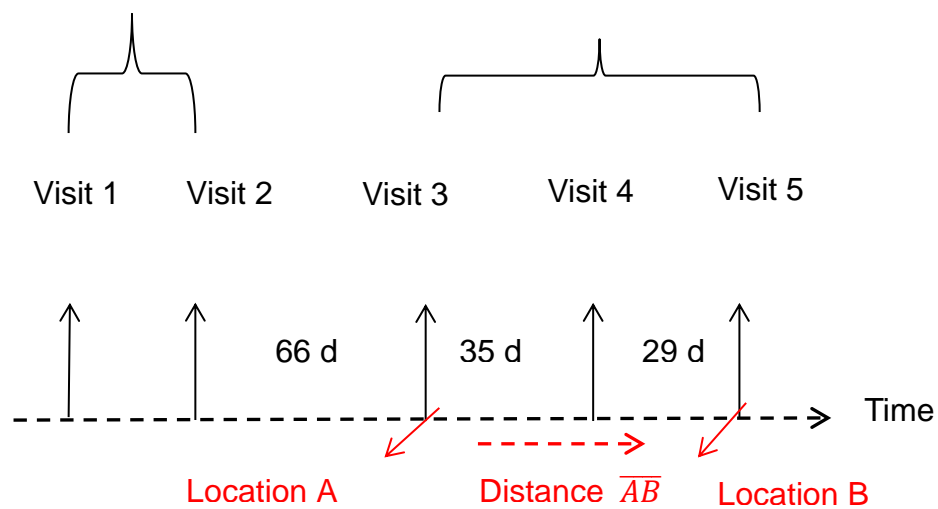

Supplement: Additional file 5: Figure S2 — Measurement of health system delay (HSD). We assumed that respiratory-related visits were of the same respiratory episode if the interval between two respiratory-related visits was ≦60 days and different episodes if >60 days when examining HSD. For example, Visit 3 was considered belonging to the same episode and included in our study because the interval between Visit 3 and Visit 4 was 35 days (≦60). Visit 2 was identified as another respiratory episode and excluded because the interval between Visit 2 and Visit 3 was 66 days (>60). $\overline{AB}$AB¯ indicates the Euclidean distance between Visit 3 and the visit where TB treatment was initialized (Visit 5). (PDF 311 kb) [file 12879_2015_1228_MOESM5_ESM.pdf]

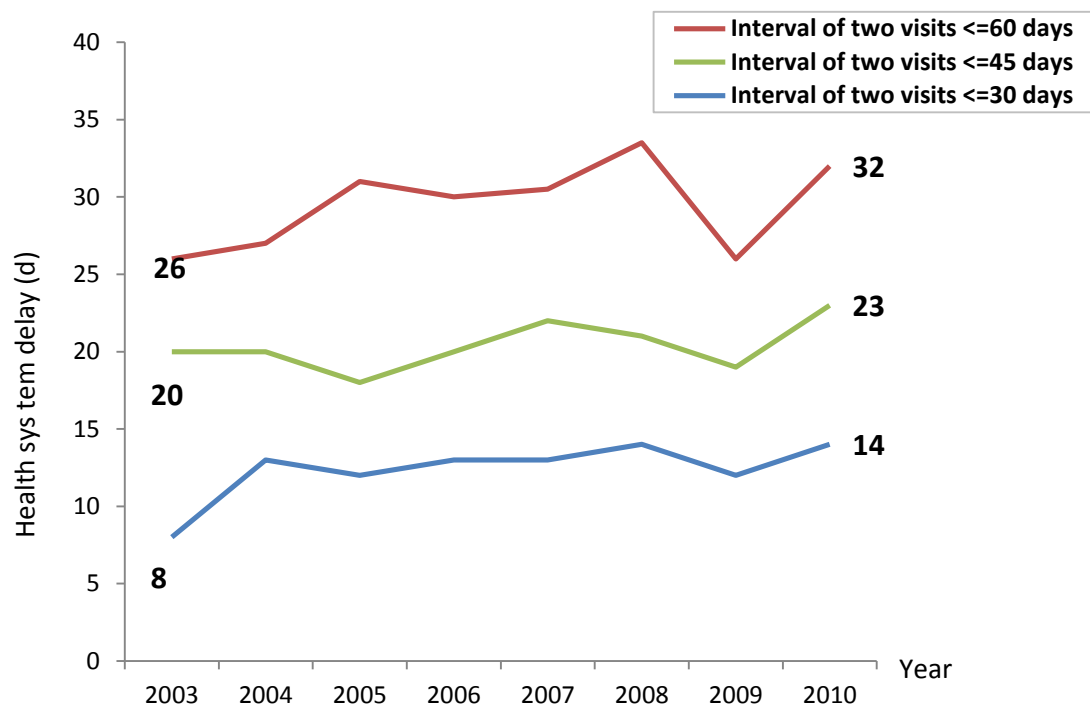

Supplement: Additional file 6: Figure S3 — Sensitivity analysis of health system delay (HSD) according to different cut-off intervals between two respiratory-related visits. (PDF 168 kb) [file 12879_2015_1228_MOESM6_ESM.pdf]

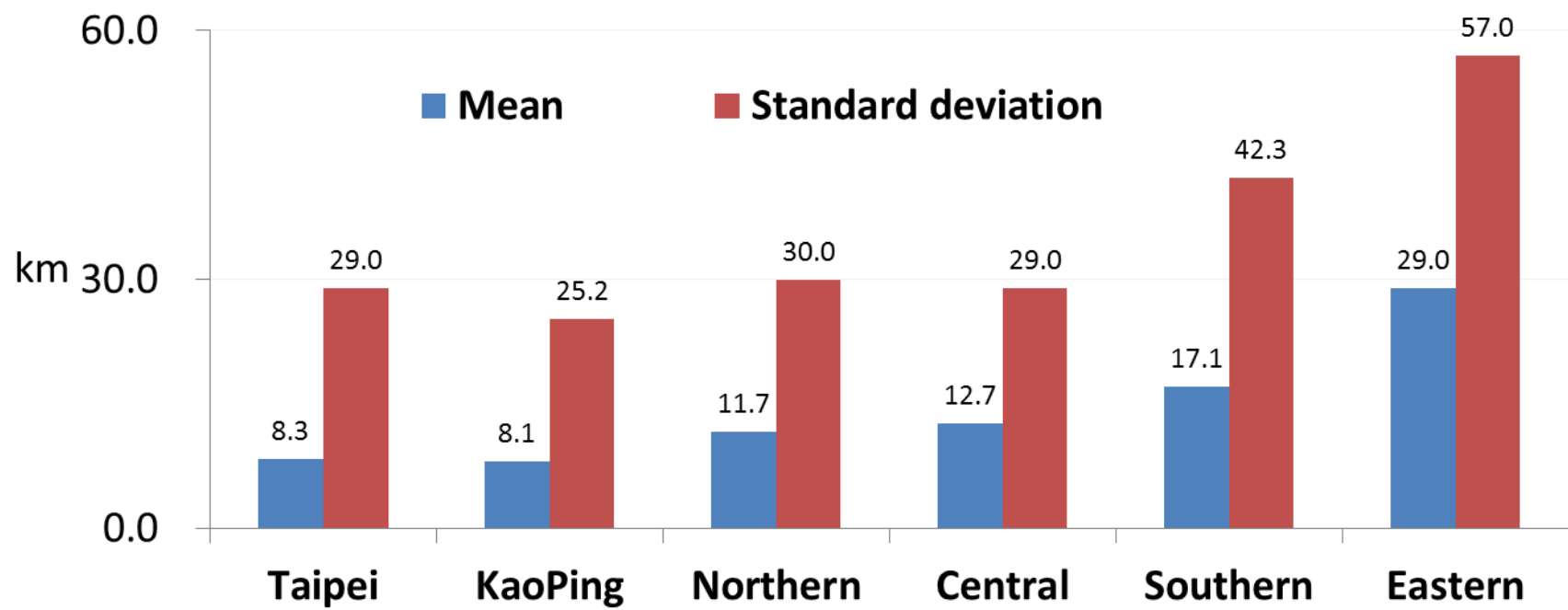

Supplement: Additional file 7: Figure S4 — Travel distance from the point of initial visit to that of the initiation of TB treatment by region. (PDF 104 kb) [file 12879_2015_1228_MOESM7_ESM.pdf]
