# Supplementary material for: Health system delay among patients with tuberculosis in Taiwan: 2003–2010
Source: BMC Infect Dis. 2015 Nov 2;15:491. doi: 10.1186/s12879-015-1228-x (PMC4629405; doi:10.1186/s12879-015-1228-x)
Supplement: Additional file 2: Table S1 — ICD-9-CM codes associated with respiratory-related visits. (PDF 191 kb) [file 12879_2015_1228_MOESM2_ESM.pdf]

Table S1 ICD-9-CM codes associated with respiratory-related visits.

Code	Disease
460	Acute nasopharyngitis
4610	Acute sinusitis, Maxillary
4611	Acute sinusitis, Frontal
4612	Acute sinusitis, Ethmoidal
4613	Acute sinusitis, Sphenoidal
4618	Other acute sinusitis
4619	Acute sinusitis, unspecified
462	Acute pharyngitis
463	Acute tonsillitis
4640	Acute laryngitis
46410	Acute tracheitis without mention of obstruction
46411	Acute tracheitis with obstruction
46420	Acute laryngotracheitis without mention of obstruction
46421	Acute laryngotracheitis with obstruction
46430	Acute epiglottitis without mention of obstruction
46431	Acute epiglottitis with obstruction
4650	Acute laryngopharyngitis
4658	Acute upper respiratory infections other multiple sites
4659	Acute upper respiratory infections, unspecified site
4660	Acute bronchitis
46611	Acute bronchiolitis due to respiratory syncytial virus
46619	Acute bronchiolitis due to other infectious organisms
4720	Chronic rhinitis
4721	Chronic pharyngitis
4722	Chronic nasopharyngitis
4730	Maxillary
4731	Frontal
4732	Ethmoidal
4733	Sphenoidal
4738	Other chronic sinusitis
4739	Unspecified sinusitis (chronic)
47400	Chronic tonsillitis
47401	Chronic adenoiditis
47402	Chronic tonsillitis and adenoiditis
47410	Hypertrophy of tonsils with adenoids

47411	Hypertrophy of tonsils alone
47412	Hypertrophy of adenoids alone
4742	Adenoid vegetations
4748	Other chronic disease of tonsils and adenoids
4749	Unspecified chronic disease of tonsils and adenoids
475	Peritonsillar abscess
4760	Chronic laryngitis
4761	Chronic laryngotracheitis
4770	Allergic rhinitis due to pollen
4771	Allergic rhinitis due to food
4778	Allergic rhinitis due to other allergen
4779	Allergic rhinitis, cause unspecified
4781	Other diseases of nasal cavity and sinuses
47820	Unspecified disease of pharynx
47821	Cellulitis of pharynx or nasopharynx
47822	Parapharyngeal abscess
47824	Retropharyngeal abscess
47825	Edema of pharynx or nasopharynx
47826	Cyst of pharynx or nasopharynx
47829	Other diseases of pharynx
47830	Paralysis, unspecified
47831	Paralysis of vocal cords or larynx Unilateral, partial
47832	Paralysis of vocal cords or larynx Unilateral, complete
47833	Paralysis of vocal cords or larynx Bilateral, partial
47834	Paralysis of vocal cords or larynx Bilateral, complete
4784	Polyp of vocal cord or larynx
4785	Other diseases of vocal cords
4786	Edema of larynx
47870	Unspecified disease of larynx
47871	Cellulitis and perichondritis of larynx
47874	Stenosis of larynx
47875	Laryngeal spasm
47879	Other diseases of larynx
4788	Upper respiratory tract hypersensitivity reaction, site unspecified
4789	Other and unspecified diseases of upper respiratory tract
4800	Pneumonia due to adenovirus
4801	Pneumonia due to respiratory syncytial virus

4802	Pneumonia due to parainfluenza virus
4808	Pneumonia due to other virus not elsewhere classified
4809	Viral pneumonia, unspecified
481	Pneumococcal pneumonia
4822	Pneumonia due to Hemophilus influenzae
48230	Pneumonia due to Streptococcus, unspecified
48231	Pneumonia due to Streptococcus Group A
48232	Pneumonia due to Streptococcus Group B
48239	Pneumonia due to Streptococcus, other Streptococcus
48240	Pneumonia due to Staphylococcus, unspecified
48241	Methicillin susceptible pneumonia due to Staphylococcus aureus
48249	Other Staphylococcus pneumonia
48281	Pneumonia due to Anaerobes
48282	Pneumonia due to Escherichia coli
48283	Pneumonia due to other gram-negative bacteria
48284	Legionnaires' disease
48289	Other specified bacteria
4829	Bacterial pneumonia unspecified
4830	Mycoplasma pneumoniae
4831	Pneumonia due to Chlamydia
4838	Pneumonia due to other specified organism
4841	Pneumonia in cytomegalic inclusion disease
4846	Pneumonia in aspergillosis
4847	Pneumonia in other systemic mycoses
4848	Pneumonia in other infectious diseases classified elsewhere
485	Bronchopneumonia, organism unspecified
486	Pneumonia, organism unspecified
4870	Influenza with pneumonia
4871	Influenza with other respiratory manifestations
4878	Influenza with other manifestations
490	Bronchitis, not specified as acute or chronic
4910	Simple chronic bronchitis
4911	Mucopurulent chronic bronchitis
49120	Obstructive chronic bronchitis without exacerbation
49121	Obstructive chronic bronchitis with (acute) exacerbation
4918	Other chronic bronchitis
4919	Unspecified chronic bronchitis

4920	Emphysematous bleb
4928	Other emphysema
49300	Extrinsic asthma
49301	Extrinsic asthma with status asthmaticus
49302	Extrinsic asthma with (acute) exacerbation
49310	Intrinsic asthma
49311	Intrinsic asthma with status asthmaticus
49312	Intrinsic asthma with (acute) exacerbation
49320	Chronic obstructive asthma
49321	Chronic obstructive asthma with status asthmaticus
49322	Chronic obstructive asthma with (acute) exacerbation
4950	Farmers' lung
4951	Bagassosis
4952	Bird-fanciers' lung
4953	Suberosis
4954	Malt workers' lung
4955	Mushroom workers' lung
4956	Maple bark-strippers' lung
4957	Ventilation pneumonitis
4958	Other specified allergic alveolitis and pneumonitis
4959	Unspecified allergic alveolitis and pneumonitis
496	Chronic airway obstruction, not elsewhere classified
500	Coal workers' pneumoconiosis
501	Asbestosis
502	Pneumoconiosis due to other silica or silicates
503	Pneumoconiosis due to other inorganic dust
504	Pneumonopathy due to inhalation of other dust
505	Pneumoconiosis, unspecified
5060	Bronchitis and pneumonitis due to fumes and vapors
5061	Acute pulmonary edema due to fumes and vapors
5062	Upper respiratory inflammation due to fumes and vapors
5063	Other acute and subacute respiratory conditions due to fumes and vapors
5064	Chronic respiratory conditions due to fumes and vapors
5069	Unspecified respiratory conditions due to fumes and vapors
5070	Pneumonitis due to inhalation of food or vomitus
5071	Pneumonitis due to inhalation of oils and essences
5078	Pneumonitis due to other solids and liquids

5080	Acute pulmonary manifestations due to radiation
5081	Chronic and other pulmonary manifestations due to radiation
5088	Respiratory conditions due to other specified external agents
5089	Respiratory conditions due to unspecified external agent
5100	Empyema with fistula
5109	Empyema without mention of fistula
5110	Pleurisy without mention of effusion or current tuberculosis
5111	Pleurisy with effusion, with mention of a bacterial cause other than tuberculosis
5118	Other specified forms of effusion, except tuberculous
5119	Unspecified pleural effusion
5120	Spontaneous tension pneumothorax
5128	Other spontaneous pneumothorax
5130	Abscess of lung
5131	Abscess of mediastinum
514	Pulmonary congestion and hypostasis
515	Postinflammatory pulmonary fibrosis
5160	Pulmonary alveolar proteinosis
5163	Idiopathic fibrosing alveolitis
5168	Other specified alveolar and parietoalveolar pneumonopathies
5169	Unspecified alveolar and parietoalveolar pneumonopathy
5171	Rheumatic pneumonia
5172	Lung involvement in systemic sclerosis
5178	Lung involvement in other diseases classified elsewhere
5180	Pulmonary collapse
5181	Interstitial emphysema
5182	Compensatory emphysema
5183	Pulmonary eosinophilia
5184	Acute edema of lung, unspecified
5185	Pulmonary insufficiency following trauma and surgery
5186	Allergic bronchopulmonary aspergillosis
51881	Acute respiratory failure
51882	Other pulmonary insufficiency, not elsewhere classified
51883	Chronic respiratory failure
51884	Acute and chronic respiratory failure
51889	Other diseases of lung, not elsewhere classified
5191	Other diseases of trachea and bronchus, not elsewhere classified
5192	Mediastinitis

5193	Other diseases of mediastinum, not elsewhere classified
5198	Other diseases of respiratory system, not elsewhere classified
5199	Unspecified disease of respiratory system
7806	Fever
78079	Other malaise and fatigue
78321	Loss of weight
78600	Respiratory abnormality, unspecified
78602	Orthopnea
78605	Shortness of breath
78606	Tachypnea
78607	Wheezing
78609	Other
7861	Stridor
7862	Cough
7863	Hemoptysis
7864	Abnormal sputum
78650	Chest pain, unspecified
78651	Precordial pain
78652	Painful respiration
78659	Other
7866	Swelling, mass, or lump in chest
7867	Abnormal chest sounds
7869	Other symptoms involving respiratory system and chest
