# Supplementary material for: Health system delay among patients with tuberculosis in Taiwan: 2003–2010
Source: BMC Infect Dis. 2015 Nov 2;15:491. doi: 10.1186/s12879-015-1228-x (PMC4629405; doi:10.1186/s12879-015-1228-x)
Supplement: Additional file 3: Table S2 — Medicines associated with respiratory-related visits. (PDF 86 kb) [file 12879_2015_1228_MOESM3_ESM.pdf]

Table S2 Medicines associated with respiratory-related visits.

Type	Medicine
Mucolytic	Acetylcysteine<br>Acetylcysteine Sodium<br>Ambroxol HCl<br>Compound Opium and Glycyrrhiza Mixture<br>Mesna<br>Cough Mixture
Antitussive agents	Codeine Phosphate<br>Dextromethorphan HBr
Bronchodilator	Albuterol Sulfate<br>Aminophylline<br>Procaterol HCl<br>Formoterol and Budesonide<br>Isoproterenol HCl<br>Pseudoephedrine HCl<br>Tiotropium
Asthma drugs	Budesonide<br>Fluticasone Propionate<br>Montelukast Sodium
Influenza drugs	Oseltamivir Phosphate

Source: http://dept.ntuh.gov.tw/phar/intranet/druginfo/index.asp?drug_code
