# Supplementary material for: Health system delay among patients with tuberculosis in Taiwan: 2003–2010
Source: BMC Infect Dis. 2015 Nov 2;15:491. doi: 10.1186/s12879-015-1228-x (PMC4629405; doi:10.1186/s12879-015-1228-x)
Supplement: Additional file 4: Table S3 — Procedures associated with respiratory-related visits. (PDF 161 kb) [file 12879_2015_1228_MOESM4_ESM.pdf]

Table S3 Procedures associated with respiratory-related visits.

Code	Procedure
13001C	Sputum routine
13012C	Acid-fast culture
13013C	Sensitivity test of acid-fast
13024C	Tuberculosis test
E3020C	Tuberculosis test or chest x-ray check consultancy
E3039C	Tuberculosis test or chest x-ray check consultancy
12106C	Tuberculin test
32001C	Chest view (including each view of chest file)
32002C	Chest view (including each view of chest file)
32003C	Chest P – A and both oblique view (with bariummeal)
33070B	Computered tomography (C.T.) without contrast
33071B	Computered tomography (C.T.) with contrast
33072B	Computered tomography (C.T.) with/without contrast
28006C	Bronchoscopy
12020B	Mycoplasma pneumonia Ab test
12126B	Streptococcus pneumonia Ag-latex
12132B	Chlamydia pneumonia Ag
12172B	Pneumococcus Ag (urine)
12191C	Legionella pneumophila Ag (urine)
12069B	Cryptococcus antigen
12179C	Aspergillus Ag
14020B	Influenza A CF Ab
14021B	Influenza B CF Ab
16003C	Pleural fluid routine

Note: Codes of procedure were from National Health Insurance Administration, Ministry of Health and Welfare, Taiwan. (http://www.nhi.gov.tw/query/query2.aspx?menu=20&menu_id=710&webdata_id=3633&WD_ID=900)
