# Supplementary material for: Health system delay among patients with tuberculosis in Taiwan: 2003–2010
Source: BMC Infect Dis. 2015 Nov 2;15:491. doi: 10.1186/s12879-015-1228-x (PMC4629405; doi:10.1186/s12879-015-1228-x)
Supplement: Additional file 8: Table S4 — Number of medical providers and tuberculosis (TB)-related providers by region in 2010. (PDF 164 kb) [file 12879_2015_1228_MOESM8_ESM.pdf]

Table S4 Number of medical providers and tuberculosis (TB)-related providers by region in Taiwan, 2010.

Region	Number of medical providers*	Number of medical providers per 10,000 people[§]	Number of TB-related[‡] medical providers	Number of TB-related medical providers per 10,000 people
Central	4,546	10.14	62	0.13
Eastern	451	7.91	12	0.21
KaoPing	3,523	9.40	47	0.12
Northern	2,592	7.42	41	0.11
Southern	2,946	8.64	38	0.11
Taipei	6,512	8.84	81	0.11
Total	20,570	8.92	281	0.12

[*]Source: Ambulatory care expenditures by visits in 2010 from the Longitudinal Health Insurance Database 2000.

[‡]Chest, Chest Surgery, TB, Infectious Disease, and Pulmonary and Critical Care specialties.

[§]Statistical Yearbook of Interior 2010, Taiwan.
